# Sociodemographic and Health-Related Factors Associated with Severity of Cognitive Impairment in Elderly Patients Hospital-ized in a Geriatric Clinic

**DOI:** 10.3390/brainsci11020170

**Published:** 2021-01-29

**Authors:** Marta Kłoszewska, Błażej Łyszczarz, Kornelia Kędziora-Kornatowska

**Affiliations:** 1 Department of Neurosurgery and Neurology, Faculty of Health Sciences, Nicolaus Copernicus University in Toruń, 85-168 Bydgoszcz, Poland; marta.kloszewska@cm.umk.pl; 2 Department of Health Economics, Faculty of Health Sciences, Nicolaus Copernicus University in Toruń, 85-830 Bydgoszcz, Poland; 3 Department of Geriatrics, Faculty of Health Sciences, Nicolaus Copernicus University in Toruń, 85-094 Bydgoszcz, Poland; kornelia.kornatowska@cm.umk.pl

**Keywords:** cognitive impairment, dementia, sociodemographic factors, risk factors

## Abstract

Identification of risk factors for cognitive impairment is crucial for providing proper care and treatment. The aim of the study was to investigate the relationship between sociodemographic and health-related factors and the severity of cognitive impairment in elderly patients. In this retrospective study, we assessed the medical documentation of 323 patients aged 60+ years hospitalized in a geriatric clinic of university hospital. The patients were classified into five groups of cognitive impairment severity based on the Mini Mental State Examination and Clock Drawing Test. Kruskal-Wallis and Chi square tests and multivariate ordinal logistic regression were used to assess relationships involved. Cognitive impairment was identified in 84.2% of subjects. The following factors were indicative for higher level of cognitive disorders: primary and vocational education, older age, presence of vascular brain injury, and inability of walking independently. On the other hand, the factors associated with lower severity of cognitive impairment were co-morbid anxiety disorders, ischemic heart disease, and a higher BMI index. Dementia is one of the leading causes of disability and mortality in the elderly. Enhancing knowledge about the risk factors that worsen cognition is particularly relevant for accelerating the diagnosis of dementia and improving patient care.

## 1. Introduction

Increasing life expectancy is leading to a worldwide rise of the elderly population. In the 37 countries of Organization for Economic Cooperation and Development, the share of people over 65 years of age in general population grew from 11.6% in 1990 to 13.1% in 2000 and 17.2% in 2018. In several developed countries this share exceeds 20%, reaching a level of 28.1% in Japan—the fastest aging society in the world [1]. A higher number of elderly people is related with the increased prevalence of chronic diseases such as dementia. According to the 2019 World Alzheimer Report, around 50 million people in the world are affected by dementia, and by 2050 this number will triple to 152 million. Worldwide, there is a new case of dementia developed every 3 s [2]. Many people affected by dementia are not appropriately diagnosed. According to recent meta-analysis [3], the prevalence of undiagnosed dementia in society is 61.7%. The detection of dementia and cognitive impairment is essential for ensuring adequate care and treatment as well as improving the quality of life, and early diagnosis improves patient care and enhances patients’ quality of life [4].

There have been many studies identifying potential risk factors for the occurrence of cognitive disorders and their progression. Cognitive functions are impacted by numerous factors such as age, education level, the presence of diseases that increase cardiovascular risk, such as diabetes, hypertension, obesity, and low physical activity [5,6].

However, people with cognitive impairment constitute a fairly diverse group. This group includes both patients with mild cognitive impairment and dementia. In the dementia group, one could distinguish different stages of the disease, from mild through moderate to severe. This classification applies to both patients with Alzheimer’s disease (AD) and those with other causes of cognitive disorders, including vascular dementia.

When evaluating risk factors or the coexistence of factors that may influence cognitive impairment, the majority of studies focus on comparing two groups, e.g., healthy patients with mild cognitive impairment (MCI), healthy patients with dementia or MCI with mild dementia. As far as we are concerned, there are no studies assessing the individual severity of cognitive disorders or a cross-sectional analysis of all stages of the disease. This work attempts to fill this gap; therefore, the aim of this study was to investigate associations of selected sociodemographic and health-related factors with severity of cognitive impairment.

## 2. Materials and Methods

### 2.1. Study Material

We assessed medical records of patients hospitalized at the Geriatrics Clinic of University Hospital No. 1 in Bydgoszcz, which is the highest reference level and the largest hospital in Kujawsko-Pomorskie, a region located in the northern Poland with a population of around 2,000,000. The data of subsequent patients admitted to the clinic between 1 January, 2018 and 30 September, 2018, were analyzed. The study inclusion criteria for clinic patients were age of 60+ years old, stable clinical condition and complete geriatric evaluation with full psychological examination.

In the period analyzed, 1055 patients were admitted to the clinic, of which 323 (30.6%) met the inclusion criteria. We have not applied a priori procedure for sample size determination because this study used data on all the clinic’s patients in the predetermined period investigated.

The dataset used for the analysis is available online as a Supplementary File (Table S1).

### 2.2. Diagnosis of Cognitive Impairment

Diagnosis and worsening of cognitive disorders were assessed based on neuropsychological examination using the Mini-Mental State Examination (MMSE) score (score range 0–30; severity of impairment classification: 0–10 severe dementia, 11–18 moderate dementia, 19–23 mild dementia, 24–26 MCI, 27–30 no cognitive impairment) adjusted for age and level of education [7] and on the Clock Drawing Test (CDT). To exclude severe depression, we used the Geriatric Depression Scale. The remaining neuropsychological tests were individually selected in accordance with generally accepted standards and the patient’s needs. Final diagnosis of cognitive disorders was made by a geriatric specialist based on the above tests and clinical condition of a patient. The cause of cognitive impairment was not distinguished. Patients with severe depression and in unstable clinical condition were excluded.

The subjects were classified into five groups according to the severity of cognitive disorders: I—no cognitive impairment (control group); II—mild cognitive impairment; III—mild dementia; IV—moderate dementia; and V—severe dementia.

For all subjects, sociodemographic data and information on comorbidities that could affect cognitive functions were collected based on the appropriate diagnosis in the patient information sheet, the results of additional tests during hospitalization and medical examination. The sociodemographic data included: sex, age (years), education level (primary, vocational, secondary, tertiary), place of residence (urban or rural), household status (living alone or with family), marital status (single/widowed or married), having a close family (yes or no). The data on health-related factors included comorbidities (depression, anxiety disorders, hypertension, diabetes, atrial fibrillation, ischemic heart disease, heart failure, obesity, hypercholesterolemia, myocardial infarction, stroke, vascular brain injury, atherosclerosis) as well as functional status (walking independence), body mass index (BMI), and deficiencies of vitamin D, vitamin B12, and folic acid. The above set of factors included in the analysis is not exhaustive. Other potential correlates of cognitive impairment such as hearing loss [8,9] or vestibular loss [10] could contribute to improved understanding of cognitive impairment severity. Unfortunately, we were not able to scrutinize these relationships because of study design (lack of information on these factors in medical records).

### 2.3. Statistical Analysis

The study used a number of statistical methods. Structure analysis was used to describe sociodemographic characteristics and distribution of cognitive disorders in the studied group. Contingency tables accompanied by Kruskal-Wallis test, chi-square test, and Cramer’s V coefficient were used to analyze the relationships between sociodemographic and health factors, and severity of cognitive impairment. Multivariate ordinal logistic regression was used to model this last relationship. We started with a regression model including all the potential covariates and backward elimination was applied with sequential elimination of covariates based on decreasing *p*-value and starting from the one with the highest *p*-value. Akaike and Schwartz information criteria were used for the purpose of final model selection. Additionally, heteroscedasticity and autocorrelation robust standard errors were used for the regression model.

Statistical significance was set up at a level of *p* < 0.05. Because this study tested numerous relationships from the same sample, we applied an adaptation of the significance level by applying the Benjamini-Hochberg procedure [11] with a false discovery rate of 0.1. This procedure was used separately for univariate (see Section 3.3, Section 3.4 and Section 3.5) and multivariate (see Section 3.6) analyses.

## 3. Results

### 3.1. General Characteristics of Study Population

The study covered a group of 323 patients over the age of 60 hospitalized in the Geriatrics Clinic of University Hospital No. 1 in Bydgoszcz. The sociodemographic characteristics of the study group are structured in Table 1.

Most of the patients were women (73.1%). The mean age of the study population was 80.5 ± 7.5 years. Most patients had secondary (41.5%) and primary (27.9%) education. Those residing in urban areas accounted for 82.0% of the entire group. Almost 2/3 of the patients lived with a family, and similar share was single or widowed (Table 1).

### 3.2. Prevalence of Cognitive Impairment

**Figure 1:**
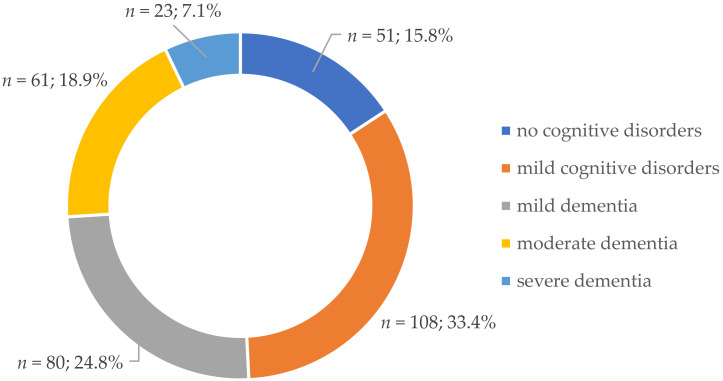
Prevalence of cognitive impairment by severity of disorders.

Cognitive impairment was found in 272 subjects (84.2%). Patients with mild cognitive impairment represented 1/3 of all subjects (33.4%). Dementia was found in slightly more than a half of all patients (50.8%), with the greatest number of patients having mild dementia (24.8%) and the least with severe dementia (7.1%). No cognitive impairment was found in 15.8% of the study group (Figure 1).

### 3.3. Severity of Cognitive Impairment and Sociodemographic Factors

Among the sociodemographic factors investigated, only age exhibited statistically significant correlation with the severity of cognitive impairment (*p* < 0.001) (Table 2).

Although other sociodemographic factors did not show statistical association with severity of cognitive impairment, it is worth noting that the share of people with severe dementia was the highest in patients with primary education (12.2%) and this share generally decreased with increased level of education. The lowest educated patients constituted 47.8% of subjects with severe dementia (group V), while the share of those with tertiary education was only 4.3% of patients in this group. Moreover, the share of patients severely affected by dementia among women (those being single or widowed) was almost twice (three times) as high as in men (married); however, these relationships proved to be insignificant (Table 2).

### 3.4. Severity of Cognitive Impairment and Comorbidities

The presence of vascular brain injury was significantly associated with the severity of cognitive impairment (*p* < 0.001). The share of patients with dementia (groups III, IV, and V) among those without vascular brain injury was 36.7%, while it was 74.7% among those affected by this disease. The strength of the relationship between being affected by vascular brain injury and the severity of cognitive impairment was the highest among all the diseases studied (Cramer’s V = 0.383). The share of patients without dementia (groups I and II) who suffered from anxiety disorders was significantly higher (71.0%) than in those without anxiety disorders (43.3%, *p* < 0.001). Additionally, the presence of ischemic heart disease was significantly associated with the severity of cognitive impairment (*p* = 0.029). The share of patients with dementia (groups III, IV, and V) among people with ischemic heart disease was 40.9%, while it was 54.2% in patients without this disease. Moreover, the other conditions significantly associated with severity of cognitive disorders were obesity (*p* = 0.025), hypercholesterolemia (*p* = 0.019), and prior myocardial infarction (*p* = 0.012) (Table 3).

### 3.5. Severity of Cognitive Impairment and Other Health-Related Factors

Considering body mass, the differences between the groups were statistically significant and, generally, those with dementia had lower BMI (*p* < 0.001). In addition, the ability to walk independently was significantly associated with severity of cognitive impairment (*p* < 0.001); among those having this ability only 3.1% (12.2%) had severe dementia (moderate dementia) while in the group of those who did not walk independently this share was 13.5% (29.4). The distribution of vitamins and folic acid deficiencies in particular cognitive impairment groups was so similar that the differences between these groups were not statistically significant (Table 4).

### 3.6. Multivariate Analysis of Factors Associated with Severity of Cognitive Impairment

The multivariate model was built using ordinal logistic regression with the dependent variable of cognitive impairment severity (five-level variable, groups I to V of severity). The final specification was based on choosing the model with a set of explanatory variables minimizing the values of Akaike and Schwartz information criteria (Table 5).

All variables in the final model were significantly associated with severity of cognitive impairment. Each additional year of age was correlated with higher odds (OR = 1.07; *p* < 0.001) of developing one more level of severe cognitive impairment. Having primary and vocational education increased the odds of being in higher category of cognitive disorders by 2.43 times (*p* = 0.003) and 2.19 times (*p* = 0.027), respectively, compared to having tertiary education. The presence of anxiety disorders (OR = 0.36; *p* < 0.001) and ischemic heart disease (OR = 0.59, *p* = 0.023) coincided with a lower probability of experiencing more severe cognitive disorders. On the other hand, those suffering from vascular brain injury (OR = 3.44; *p* < 0.001) and that were unable to walk independently (OR = 2.36; *p* < 0.001) had a significantly greater odds of being in a higher category of cognitive impairment. Finally, higher BMI was correlated with a lower probability of more severe cognitive disorders (OR = 0.947; *p* = 0.001).

## 4. Discussion

This study evaluated the associations between selected sociodemographic and health-related characteristics and the severity of cognitive impairment based on retrospective data on 323 patients hospitalized in a geriatric clinic of university hospital in Bydgoszcz, Poland. The novelty of this research lays in the fact that not only the presence but also the severity of cognitive disorders was investigated for a possible association with a range of factors.

Cognitive disorders are a major problem of ageing societies. Recent studies from high income countries show that while the incidence of dementia is steadily increasing (mainly due to population aging) the risk of dementia is decreasing. This trend could assist in lowering the expected rise in morbidity resulting from the growing number of elderly people. The main causes of this reduced risk of dementia could be the growing level of education and aggressive treatment of key cardiovascular risk factors such as hypertension and hypercholesterolemia [12]. Langa et al. [13] compared data from the United States on the incidence of dementia in people aged 65 and over in 2000 and 2012 and found that this incidence decreased from 11.6% in 2000 to 8.8% in 2012 (*p* <0.001), and a greater number of years of education was associated with a lower risk of dementia. Similarly, a Swedish study [14] on the incidence of dementia between 1987 and 2016 showed that it began to decline over the last five years of the study. The largest decrease was recorded in people aged 70 to 74 (−5.5%), followed by 75 to 79-year-olds by 4.5% and in 80 to 84-year-olds by 4%. This decrease affected both genders and all educational levels. The question remains open whether this positive trend will be sustained in future when global levels of obesity and diabetes are rising, and whether this trend also applies to low- and middle-income countries. These are key questions without answers, which will have substantial magnitude in the decades to come [12].

Taking into account the increasing prevalence of cognitive disorders, it is crucial to identify factors that may affect the presence and severity of these disorders over time, and if those factors could have a protective effect.

Age is a recognized risk factor for the development of cognitive impairment [15,16]. Our research confirmed a notable association between age and severity of cognitive disorders, which is in line with previous research. In study [17], patients with dementia and MCI were significantly older than those without cognitive impairment, but no differences were found between the group of patients with MCI and AD. On the other hand, in [18] patients with AD were significantly older than the group with MCI and those without cognitive impairment. In our study, education had a considerable impact on being included in the higher category of cognitive disorders. Compared to tertiary education, primary and vocational education increased the odds of one level more of severe cognitive impairment by more than twice. This result is consistent with numerous studies, showing the protective effect of education on the risk of developing cognitive disorders. For example, the study of a population of over 4800 people aged over 65 demonstrated, that the number of years of education has a major protective impact on cognitive functions [19]. In addition, people with MCI and AD had fewer years of education than healthy people, whereby the differences were statistically significant only in the comparison of the healthy groups with MCI and AD, and no differences were found between the MCI and AD groups [17].

Vascular risk factors are believed to expose the progression of cognitive impairment and the transition from MCI to dementia [20,21]. This study investigated which diseases coexist with the severity of cognitive disorders. The results show that hypertension was the most common disease among our subjects. Yet, the differences between patients with different levels of cognitive impairment were statistically insignificant (after applying the Benjamini-Hochberg procedure) and the final regression model showed no effect of hypertension on the severity of cognitive impairment. Our study suggests that people suffering from vascular brain injury have higher odds of experiencing more severe cognitive disorders. The literature identifies that cerebrovascular changes, such as hemorrhagic infarctions, small and large ischemic cortical infarcts, vasculopathies, and white matter lesions, increase the risk of dementia, but specific underlying causes remain unclear. Strokes or changes in white matter can directly damage areas of the brain important for memory function, such as the thalamus and corticothalamic projections. They may also increase β-amyloid deposition, which in turn may lead to cognitive decline or may induce inflammatory responses that impair cognition [22].

Mood disorders are one of the most common anomalies in the elderly. It is estimated that the prevalence of depression in people over 60 years of age ranges from 15% to 20%, and among older people using medical care, even up to 30% [23]. In our research, depressive disorders were found in 38.4% of patients. The correlation between late-onset depression and dementia has not been clarified, and there is an ongoing debate in the literature as to whether depression is a prodrome of dementia or a risk factor for the development of dementia [24]. Some studies report that depressive disorders often appear at the beginning of the dementia process and as it progresses, the frequency of depression increases [25]. That said, the opinion that depressive disorders are most typical for mild dementia is much more widespread. Depression is the most common in mild dementia syndromes and its prevalence is estimated at 20% to 40% [26]. As the severity of dementia increases, the number of depressive symptoms decrease, and they are more difficult to assess. Depression of mild intensity occurs in approximately 33% of patients with mild dementia, in 23% of patients with moderate dementia and in 12% of those with severe dementia [27]. In our research, we found that depression was more common in people with mild dementia than with moderate and severe dementia. However, these differences were not significant.

Anxiety disorders are common in old age and, according to various estimates, affect 3.2% to 21.6% of the population [28]. Anxiety symptoms in the elderly can cause memory problems, increase the risk of physical disability, lower their quality of life and increase the risk of death. In 60% of patients, anxiety symptoms coexist with depressive disorders [29]. There is little data on the correlation of anxiety disorders with the severity of cognitive impairment. A meta-analysis [30] showed that anxiety symptoms occur in 8% to 71% of people with dementia and in 10% to 74% of people with MCI. In our research, anxiety disorders were observed in 29.6% of patients with MCI, in 16.2% with mild dementia, in 9.8% with moderate dementia and in 4.3% with severe dementia. The regression model demonstrated that the presence of anxiety disorders was associated with lower odds of being classified in the group with more severe cognitive disorders. This may result from the fact that anxiety disorders are more common in younger than in older people [29], and usually cognitive impairment increases with age. The less frequent anxiety disorders along with the worsening of cognitive disorders may also stem from diagnostic difficulties. As dementia intensifies, patients experience weakening verbal contact and might not understand questions asked and instructions, which hinders the performance of appropriate psychological tests and diagnosis of both anxiety and depression.

Considering the body mass index of patients, higher BMI was correlated with a lower probability of having more severe cognitive disorders and this outcome is consistent with a recent study of geriatric patients’ cognitive impairment [31]. Dye et al. [32] confirm that obesity is associated with impairment of cognitive functions, their accelerated deterioration and dementia in later life. In turn, another prospective study correlated both low, as well as high body mass with increased risk of cognitive impairment and AD and suggested a U-shaped relationship dependent on the age at which body weight is measured [33]. Low BMI in those suffering from dementia is a sign of disease severity and a consequence of malnutrition being typical in advanced stages of disease. On the other hand, high BMI is one of the risk factors of dementia because it is associated with cardiovascular diseases (e.g., hypertension) and obstructive sleep apnea [34] which can both contribute to cognitive decline. There is also evidence of an inverse causal relationship in the years leading up to the onset of dementia due to weight loss from malnutrition in prodromal dementia [35,36].

In recent years, there has been growing interest in the influence of vitamin deficiencies on the risk of cognitive disorders. The largest focus was on the role of vitamin D, but the conclusions from the previous studies are ambiguous. Some studies in the elderly population confirm that vitamin D deficiency is associated with an increased risk of dementia [37,38,39]. On the other hand, other research points to a lack of association between vitamin D deficiency and the risk of cognitive impairment [40,41]. Although in our research vitamin D deficiency was common, no relationship was found between the deficiency of this vitamin and the severity of cognitive disorders. There was also no such association with vitamin B12 deficiency and folic acid deficiency.

Before concluding, we shall acknowledge the limitations of our analysis. First, the study used data from a single geriatric clinic of a university hospital, and our conclusions might not be fully applicable to other populations of elderly people. Secondly, because we relied on retrospective documentation analysis, the findings are prone to typical disadvantages of this study design, including the fact that the scope of the analysis is limited to the available data. Particularly, we were not able to scrutinize the impact of factors as hearing loss or vestibulopathy on cognitive impairment severity. Additionally, the recent study on oropharyngeal dysphagia suggests that this condition may be a prodromal symptom of MCI or mild dementia and heavily modifies quality of life [42]. In addition, this factor could be included in our analysis; unfortunately, we were not able to do so because of the study design limitations. Thirdly, patient classification to severity of cognitive impairment groups is partly based on the subjective assessment of the geriatrist and this might be a source of some bias. Finally, sample size has not been determined prior to investigation. However, we included all the potential subjects into our analysis, therefore, no more patients could have been added up to increase sample size in the predetermined period investigated.

## 5. Conclusions

Cognitive impairment, particularly dementia, is one of the leading causes of disability and mortality in the elderly. This study provided evidence on the association between sociodemographic and health-related factors and severity of cognitive impairment. Such an analysis is important because it enhances knowledge on these risk factors and as such is of relevance for accelerating the diagnosis of dementia and improving patient care.

## Figures and Tables

**Table 1:** Sociodemographic characteristics of the study group.

Variable	Variant	*n*	Share
Sex	women	236	73.1%
	men	87	26.9%
Age (mv = 80.5; min = 60; max = 95; sd = 7.5)	60–74 year	72	22.3%
	75–89 year	226	70.0%
	90+ years	25	7.7%
Education level	primary	90	27.9%
	vocational	56	17.3%
	secondary	134	41.5%
	tertiary	43	13.3%
Residence	urban area	265	82.0%
	rural area	58	18.0%
Household status	living alone	117	36.2%
	living with family	204	63.2%
	living in nursing home	2	0.6%
Marital status	single/widowed	197	61.0%
	married	126	39.0%

Notes: *n*—number; mv—mean value; min—minimum value; max—maximum value; sd—standard deviation.

**Table 2:** Associations between sociodemographic factors and severity of cognitive impairment.

Variable	Category	Severity of Cognitive Impairment					Test Statistic (*p*)
		None (I)	Mild Cognitive Impairment (II)	Mild Dementia (III)	Moderate Dementia (IV)	Severe Dementia (V)	
		*n* (%)/mv ± sd *	*n* (%)/mv ± sd *	*n* (%)/mv ± sd *	*n* (%)/mv ± sd *	*n* (%)/mv ± sd *	
Age		75.5 ± 8.0	78.8 ± 8.1	82.8 ± 5.8	82.7 ± 5.9	84.9 ± 4.7	H = 43.45 (*p* < 0.001)^BH^
Sex	women	36 (15.3%)	76 (32.3%)	62 (26.3%)	43 (18.2%)	19 (8.1%)	χ^2^ = 2.63 (*p* = 0.622) V = 0.090
	men	15 (17.2%)	32 (36.8%)	18 (20.7%)	18 (20.7%)	4 (4.6%)	
Education	primary	6 (6.7%)	27 (30.0%)	28 (31.1%)	18 (20.0%)	11 (12.2%)	χ^2^ = 19.14 (*p* = 0.085) V = 0.140
	vocational	11 (19.6%)	15 (26.8%)	12 (21.4%)	15 (26.8%)	3 (5.4%)	
	secondary	24 (17.9%)	50 (37.3%)	30 (22.4%)	22 (16.4%)	8 (6.0%)	
	tertiary	10 (23.3%)	16 (37.2%)	10 (23.3%)	6 (14.0%)	1 (2.3%)	
Residence	urban area	43 (16.2%)	87 (32.8%)	65 (24.5%)	51 (19.3%)	19 (7.2%)	χ^2^ = 0.48 (*p* = 0.975) V = 0.039
	rural area	8 (13.8%)	21 (36.2%)	15 (25.9%)	10 (17.2%)	4 (6.9%)	
Household status	living alone	21 (17.8%)	35 (29.9%)	35 (29.9%)	20 (17.1%)	6 (5.1%)	χ^2^ = 4.52 (*p* = 0.340) V = 0.118
	living with family **	30 (14.6%)	73 (35.4%)	45 (21.8%)	41 (19.9%)	17 (8.3%)	
Marital status	single/widowed	29 (14.7%)	58 (29.4%)	50 (25.4%)	41 (20.8%)	19 (9.6%)	χ^2^ = 8.36 (*p* = 0.079) V = 0.161
	married	22 (17.5%)	50 (39.7%)	30 (23.8%)	20 (15.9%)	4 (3.2%)	

Notes: *n*—number; %—share in total population; mv—average; sd—standard deviation; H—statistics of Kruskal-Wallis test; χ^2^—statistic of chi-square test; V—value of Cramer’s V coefficient; BH—test value is statistically significant with the Benjamini-Hochberg procedure applied. *—for quantitative variables, m and sd values are reported, for qualitative variables—*n* and %; **—this category also includes those living in nursing homes (*n* = 2). Percentages sum up to a total in table rows and may not add up to 100% due to rounding.

**Table 3:** Associations between occurrence of comorbidities and severity of cognitive impairment.

Variable		Severity of Cognitive Impairment					Test Statistic (*p*)
		None (I)	Mild Cognitive Impairment (II)	Mild Dementia (III)	Moderate Dementia (IV)	Severe Dementia (V)	
		*n* (%)/mv ± sd *	*n* (%)/mv ± sd *	*n* (%)/mv ± sd *	*n* (%)/mv ± sd *	*n* (%)/mv ± sd *	
Depression	yes	21 (16.8%)	43 (34.4%)	38 (30.4%)	17 (13.6%)	6 (4.8%)	χ^2^ = 7.36 (*p* = 0.118) V = 0.151
	no	30 (15.2%)	65 (32.8%)	42 (21.2%)	44 (22.2%)	17 (8.6%)	
Anxiety disorders	yes	17 (24.6%)	32 (46.4%)	13 (18.8%)	6 (8.7%)	1 (1.5%)	χ^2^ = 18.78 (*p* < 0.001)^BH^ V = 0.241
	no	34 (13.4%)	76 (29.9%)	67 (26.4%)	55 (21.7%)	22 (8.7%)	
Hypertension	yes	36 (14.5%)	92 (37.0%)	64 (25.7%)	42 (16.9%)	15 (6.0%)	χ^2^ = 9.79 (*p* = 0.044) V = 0.174
	no	15 (20.3%)	16 (21.6%)	16 (21.6%)	19 (25.7%)	8 (10.8%)	
Diabetes	yes	17 (14.7%)	38 (32.8%)	28 (24.1%)	25 (21.6%)	8 (6.9%)	χ^2^ = 0.90 (*p* = 0.925) V = 0.053
	no	34 (16.4%)	70 (33.8%)	52 (25.1%)	36 (17.4%)	15 (7.3%)	
Atrial fibrillation	yes	7 (9.9%)	20 (28.2%)	22 (31.0%)	15 (21.1%)	7 (9.9%)	χ^2^ = 5.40 (*p* = 0.248) V = 0.129
	no	44 (17.5%)	88 (34.9%)	58 (23.0%)	46 (18.3%)	16 (6.4%)	
Ischemic heart disease	yes	11 (13.3%)	38 (45.8%)	21 (25.3%)	8 (9.6%)	5 (6.0%)	χ^2^ = 10.81 (*p* = 0.029)^BH^ V = 0.183
	no	40 (16.7%)	70 (29.2%)	59 (24.6%)	53 (22.1%)	18 (7.5%)	
Heart failure	yes	7 (12.5%)	16 (28.6%)	16 (28.6%)	14 (25.0%)	3 (5.4%)	χ^2^ = 2.98 (*p* = 0.562) V = 0.096
	no	44 (16.5%)	92 (34.5%)	64 (24.0%)	47 (17.6%)	20 (7.5%)	
Obesity	yes	14 (14.7%)	41 (43.2%)	23 (24.2%)	16 (16.8%)	1 (1.1%)	χ^2^ = 11.17 (*p* = 0.025)^BH^ V = 0.186
	no	37 (16.2%)	67 (29.4%)	57 (25.0%)	45 (19.7%)	22 (9.7%)	
Hypercholesterolemia	yes	23 (14.3%)	60 (37.3%)	46 (28.6%)	27 (16.8%)	5 (3.1%)	χ^2^ = 11.77 (*p* = 0.019)^BH^ V = 0.191
	no	28 (17.3%)	48 (29.6%)	34 (21.0%)	34 (21.0%)	18 (11.1%)	
Myocardial infarction	yes	1 (2.7%)	17 (46.0%)	6 (16.2%)	12 (32.4%)	1 (2.7%)	χ^2^ = 12.93 (*p* = 0.012)^BH^ V = 0.200
	no	50 (17.5%)	91 (31.8%)	74 (25.9%)	49 (17.1%)	22 (7.7%)	
Stroke	yes	7 (12.7%)	15 (27.3%)	16 (29.1%)	13 (23.6%)	4 (7.3%)	χ^2^ = 2.44 (*p* = 0.655) V = 0.087
	no	44 (16.4%)	93 (34.7%)	64 (23.9%)	48 (17.9%)	19 (7.1%)	
Vascular brain injury	yes	14 (11.6%)	17 (14.1%)	40 (33.1%)	38 (31.4%)	12 (9.9%)	χ^2^ = 47.48 (*p* < 0.001)^BH^ V = 0.383
	no	37 (18.3%)	91 (45.1%)	40 (19.8%)	23 (11.4%)	11 (5.5%)	
Atherosclerosis	yes	20 (13.2%)	49 (32.2%)	43 (28.3%)	30 (19.7%)	10 (6.6%)	χ^2^ = 3.05 (*p* = 0.550) V = 0.097
	no	31 (18.1%)	59 (34.5%)	37 (21.6%)	31 (18.1%)	13 (7.6%)	
Number of comorbidities		4.43 ± 2.26	5.03 ± 2.35	5.23 ± 2.37	4.77 ± 2.53	3.83 ± 1.92	H = 7.82 (*p* = 0.098)

Notes: *n*—number; %—share in total population; mv—average; sd—standard deviation; H—statistics of Kruskal-Wallis test; χ^2^—statistic of chi-square test; V—value of Cramer’s V coefficient; BH—test value is statistically significant with the Benjamini-Hochberg procedure applied. *—for quantitative variables, m and sd values are reported, for qualitative variables—*n* and %; Percentages sum up to a total in table rows and may not add up to 100% due to rounding.

**Table 4:** Associations between selected health-related factors and severity of cognitive impairment.

Variable		Severity of Cognitive Impairment					Test Statistic (*p*)
		None (I)	Mild Cognitive Impairment (II)	Mild Dementia (III)	Moderate Dementia (IV)	Severe Dementia [V]	
		*n* (%)/mv ± sd *	*n* (%)/mv ± sd *	*n* (%)/mv ± sd *	*n* (%)/mv ± sd *	*n* (%)/mv ± sd *	
BMI		28.2 ± 4.7	29.1 ± 5.3	26.7 ± 5.8	26.9 ± 5.8	23.5 ± 4.1	H = 27.13 (*p* < 0.001)^BH^
Functional status-independent walking	yes	40 (20.3%)	77 (39.1%)	50 (25.4%)	24 (12.2%)	6 (3.1%)	χ^2^ = 35.21 (*p* < 0.001)^BH^ V = 0.330
	no	11 (8.7%)	31 (24.6%)	30 (23.8%)	37 (29.4%)	17 (13.5%)	
Vit. D deficiency	yes, severe	27 (17.5%)	48 (31.2%)	37 (24.0%)	28 (18.2%)	14 (9.1%)	χ^2^ = 7.27 (*p* = 0.508) V = 0.106
	yes, moderate	13 (13.5%)	38 (39.6%)	19 (19.8%)	20 (20.8%)	6 (6.3%)	
	no	11 (15.1%)	22 (30.1%)	24 (32.9%)	13 (17.8%)	3 (4.1%)	
Vit. B12 deficiency	yes	14 (18.7%)	19 (25.3%)	19 (25.3%)	16 (21.3%)	7 (9.3%)	χ^2^ = 3.42 (*p* = 0.489) V = 0.103
	no	37 (14.9%)	89 (35.9%)	61 (24.6%)	45 (18.2%)	16 (6.5%)	
Folic acid deficiency	yes	16 (12.9%)	40 (32.3%)	29 (23.4%)	27 (21.8%)	12 (9.7%)	χ^2^ = 4.04 (*p* = 0.401) V = 0.112
	no	35 (17.6%)	68 (34.2%)	51 (25.6%)	34 (17.1%)	11 (5.5%)	

Notes: *n*—number; %—share in total population; mv—average; sd—standard deviation; H—statistics of Kruskal-Wallis test; χ^2^—statistic of chi-square test; V—value of Cramer’s V coefficient; BH—test value is statistically significant with the Benjamini-Hochberg procedure applied. *—for quantitative variables, m and sd values are reported, for qualitative variables—*n* and. Percentages sum up to a total in table rows and may not add up to 100% due to rounding.

**Table 5:** Ordinal logistic regression results of model for factors related to severity of cognitive impairment.

Specification	Dependent Variable: Severity of Cognitive Impairment		
	Odds Ratio	95% CI	*p*
Age	1.074	1.044; 1.105	<0.001^BH^
Education (tertiary)			
secondary	1.472	0.804; 2.580	0.177
vocational	2.192	1.095; 4.385	0.027^BH^
primary	2.431	1.347; 4.386	0.003^BH^
Anxiety disorders	0.364	0.221; 0.598	<0.001^BH^
Ischemic heart disease	0.588	0.372; 0.930	0.023^BH^
Vascular brain injury	3.438	2.120; 5.576	<0.001^BH^
Functional status (walks independently)	2.360	1.490; 3.737	<0.001^BH^
BMI	0.947	0.916; 0.978	0.001^BH^
*n* = 323; Likelihood ratio: −420.8; χ^2^ = 102.9; *p* < 0.001; Pseudo *R*^2^ = 0.135			

Notes: 95% CI—95% confidence interval; *p*—statistical significance; *n*—number of observations; BH—test value is statistically significant with the Benjamini-Hochberg procedure applied. Heteroscedasticity and autocorrelation robust standard errors were used. For categorical variables, the reference category is given in parentheses. In the case of variables illustrating the diseases, the reference category was the absence of the disease.

## Data Availability

All the data is provided in the text and Supplementary Materials.

## Supplementary Materials

The following are available online at https://www.mdpi.com/2076-3425/11/2/170/s1, Table S1: Dataset.
